# High Chromosome Number in hematological cancer cell lines is a Negative Predictor of Response to the inhibition of Aurora B and C by GSK1070916

**DOI:** 10.1186/1479-5876-9-110

**Published:** 2011-07-15

**Authors:** Christopher Moy, Catherine A Oleykowski, Ramona Plant, Joel Greshock, Junping Jing, Kurtis Bachman, Mary Ann Hardwicke, Richard Wooster, Yan Degenhardt

**Affiliations:** 1 GlaxoSmithKline Oncology Research, Cancer Metabolism, 1250 Collegeville Road, Collegeville, PA 19426, USA

## Abstract

**Background:**

Aurora kinases play critical roles in mitosis and are being evaluated as therapeutic targets in cancer. GSK1070916 is a potent, selective, ATP competitive inhibitor of Aurora kinase B and C. Translation of predictive biomarkers to the clinic can benefit patients by identifying the tumors that are more likely to respond to therapies, especially novel inhibitors such as GSK1070916.

**Methods:**

59 Hematological cancer-derived cell lines were used as models for response where *in vitro *sensitivity to GSK1070916 was based on both time and degree of cell death. The response data was analyzed along with karyotype, transcriptomics and somatic mutation profiles to determine predictors of response.

**Results:**

20 cell lines were sensitive and 39 were resistant to treatment with GSK1070916. High chromosome number was more prevalent in resistant cell lines (p-value = 0.0098, Fisher Exact Test). Greater resistance was also found in cell lines harboring polyploid subpopulations (p-value = 0.00014, Unpaired t-test). A review of NOTCH1 mutations in T-ALL cell lines showed an association between NOTCH1 mutation status and chromosome number (p-value = 0.0066, Fisher Exact Test).

**Conclusions:**

High chromosome number associated with resistance to the inhibition of Aurora B and C suggests cells with a mechanism to bypass the high ploidy checkpoint are resistant to GSK1070916. High chromosome number, a hallmark trait of many late stage hematological malignancies, varies in prevalence among hematological malignancy subtypes. The high frequency and relative ease of measurement make high chromosome number a viable negative predictive marker for GSK1070916.

## Background

Aurora kinases are an evolutionarily conserved protein family required for a variety of mitotic functions including chromosomal segregation, cell division events, and cytokinesis. Aurora Kinase B (AURKB) is a serine/threonine kinase and a component of the chromosome passenger complex (CPC) responsible for regulation of cytokinesis during mitosis. Aurora B localizes to the centromeres during prometaphase and to the spindle midphase region during anaphase onset to form a complex with survivin and the inner centromere protein (INCENP) for regulation and activation [1]. Aurora C is closely related to Aurora B with overlapping functions and similar localization patterns [2].

Aurora kinases are overexpressed in both solid and hematological malignancies [3-8] and Aurora A (AURKA) has been reported amplified in numerous malignancies [9-11]. Since Aurora kinases are exclusively expressed in proliferating cells, Aurora B inhibitors are anticipated to have reduced side effects such as neurotoxicity commonly associated with chemotherapies affecting tubulin in non-dividing cells (e.g. taxanes, vinca alkaloids). These features make Aurora kinases attractive cancer targets for therapeutics and multiple Aurora kinase inhibitors are currently being studied in early phase I and II trials [12].

GSK1070916 is a selective inhibitor of AURKB/C and has demonstrated anti-proliferative characteristics *in vitro *and *in vivo *for both solid tumors as well as hematological malignancies [13-15]. For many hematological malignancies, few treatment alternatives have been developed in recent years, and for many tumor subtypes such as Acute Myeloid Leukemia (AML) and Non-Hodgkin's Lymphoma (NHL), significant challenges remain. As with solid tumors, identification of predictive biomarkers can accelerate the clinical development of therapies for hematological malignancies through the identification of the tumors most likely to respond. One successful story of predictive biomarkers for hematological malignancies is Imatinib (Gleevec) and the BCR-ABL translocation commonly found in Chronic Mylogenous Leukemia (CML).

Here, we report the evaluation of 67 hematological tumor cell lines to identify predictive biomarkers for GSK1070916. The cell line response data was compared to the mutation patterns in the cell lines, gene expression patterns and the karyotypes of the cell lines. High chromosome number in the cell lines was associated with resistance to GSK1070916. Furthermore, treatment with GSK1070916 generally elicited a polyploidy phenotype in the hematological cell lines; as has been seen with Aurora B inhibitors. Conveniently, it is standard clinical practice to perform karyotyping on hematological cancer cells and chromosome number can serve as a resistance marker for patient response to GSK1070916.

## Methods

### Cell Line Panel

Cell lines were purchased from the American Type Culture Collection [ATCC] and the German Resource Centre for Biological Material [DSMZ] and grown to standard culture media recommended by the vendor. The majority of the cell lines were used within 6 months of acquisition and no re-authentication was performed. For the DSMZ cell bank STR DNA typing is performed for authentication and numerous authentication tests are performed at the ATCC cell bank (STR, Sequencing, SNP fingerprinting). Four cell lines in the panel (PLB-985, SKO-007, J.RT3-T3.5, CEM/C1) were excluded from analyses (data still provided in Additional File 1) since they are subclones derived from parental cell lines already on the panel (HL-60, U266, Jurkat, CCRF-CEM). There are also four cell lines (GA10, 1A2, TO 175.t, HUNS1) that are commercially available but not been published as new cell lines so their characterization may be incomplete. Cell cycle rates (doubling times) are also provided for each cell line [Additional File 1, Table S1].

### Cellular Proliferation Assays

Cells were seeded in 96-well white flat bottom plates (NUNC #136102) in the recommended growth media and incubated at 37°C in 5% CO2 overnight. The following day, 2-fold serial dilutions of GSK1070916 starting at 10 or 20 μM for a 20 point titration curve were added to the cell plates. The final DMSO concentration in all wells was 0.2%. At the time of compound addition, one set of cell plates was treated with CellTiter-Glo (Promega #G7573) to determine the number of cells present at the start of the treatment (T = 0). Following 6-7 day incubation with GSK1070916, CellTiter-Glo reagent was added using a volume equivalent to the cell culture volume in the wells. Plates were shaken and incubated at room temperature for approximately 30 minutes and the chemiluminescent signal determined using the Envison 2100 (Perkin Elmer). For analysis of cell growth inhibition, the data was plotted as the percent of the DMSO-treated control samples and the data was fit using the IDBS XLfit4 software for data analysis. Values from wells with no cells were subtracted from all samples for background correction.

### Cell Cycle Analysis

Cells were seeded in 96-well plates in the recommended growth media and incubated at 37°C in 5% CO2 overnight. The following day, three fold serial dilutions from 556 nM to 7 nM of GSK1070916 were added and the plates incubated for 24, 48 and 72 hours. After compound treatment, the cells were processed for cell cycle analysis using the detergent-trypsin Vindelov method (Vindeløv, 1983). Briefly, the treated cells were washed with PBS and suspended in 25 μl of citrate buffer (40 mM Trisodium Citrate, 250 mM Sucrose, 5% DMSO, pH 7.6) for 2 minutes. Next 100 μl of Solution A (0.03 mg/ml Trypsin, 3.4 mM Trisodium Citrate, 0.5 mM Tris Base, 0.1% NP40, 0.522 mg/ml spermine) was added followed by the addition of 100 ul of solution B (0.5 mg/ml Trypsin inhibitor (Sigma T-9003), 0.1 mg/ml of Rnase A, 3.4 mM Trisodium Citrate, 0.5 mM Tris Base, 0.1% NP40, 0.522 mg/ml spermine) for 10 minutes. The samples were then stained with the addition of 100 μl of Solution C (0.208 mg/ml propidium iodide, 1.682 mg/ml spermine, 3.4 mM Trisodium Citrate, 0.5 mM Tris Base, 0.1% NP40) for 10 minutes in the dark. These steps were all performed at room temperature while slowly shaking. The stained samples were analyzed for their DNA content using a BD Biosciences FACScan Cytometer. For each sample 3000 events were acquired on the BD Bioscience FAScan flow cytometer and no gating was applied. The instrument settings were applied so that the 2N-DNA peak on FL2-area histogram for each DMSO treated cell line was aligned at 200 fluorescent units. FL2-Area histograms were used to determine DNA content and analyzed using FlowJo software (Tree Star) which incorporates the Watson pragmatic algorithm. Histograms were plotted as number of cellular events versus FL-2-Area. DNA content was divided into 5 regions, sub-2N DNA, 2N DNA, 2N to 4N DNA, 4N DNA and >4N DNA and the percentage of cellular events in each of the five regions quantified.

### Defining Cell Sensitivity

An analysis of cell line sensitivity to GSK1070916 was performed with the data generated from screening cell lines in cellular proliferation assays and from cell cycle analyses. Cell lines were classified into one of three categories based on the time when the majority of cells contained sub-2N DNA (cell death) as determined by cell cycle analysis. "Early" responders were defined as cell lines in which the majority of cells contained sub-2N DNA within 48 hours after compound treatment, "intermediate" required a 72 hour exposure, and "late" responders required greater than or equal to a 96 hour exposure with GSK1070916 for the majority of cells to contain sub2N DNA. Furthermore, the Ymin (minimum response of the dose response curve) and the T = 0 values (the number of cells at Time zero) were determined from the cellular proliferation assays with GSK1070916. Ymin values represent the bottom of the response curve and define the largest effect of the compound. These Ymin values are evaluated relative to the number of cells at time zero using a Ymin/T0 ratio. Response curves with values significantly below 1.0 are considered cytotoxic while those above 1.0 are considered cytostatic. Using the cell cycle response data and the Ymin/T0 ratios, ""Sensitive"cell lines were defined as cell lines which were classified as an "early" or "moderate" responders to GSK1070916 treatment by cell cycle analysis (FACs) with a Ymin/T0 ratio of ≤ 0.5. Cell lines were classified as "Resistant" if they were "late" responders as defined by the cell cycle analysis and had Ymin/T = 0 ratios of > 0.5. Cell lines that were discordant between the two measures were considered ambiguous and excluded from the analysis. EC50 values greater than 500 were considered "resistant" regardless of cell cycle or Ymin values. [Additional File 1, Table S1]

### Karyotype and Mutation Data

Karyotype data included both G-banding and Spectral Karytoyping (SKY) was collected from a variety of public sources including the DSMZ [16], ATCC [17], and the NCBI Sky collection [18]. These data contain important karyotype information such chromosomal rearrangements, chromosomal additions and deletions, translocations, modality (chromosome number) and other notable structural changes in the genome. Karyotypes were compiled with response profiles from GSK1070916 and reviewed for potential biomarker candidates. [Additional File 1, Table S2]. Somatic mutation profiles for genes implicated in tumorigenesis were collected from the Catalogue of Somatic Mutations in Cancer (COSMIC)[19] and are presented in Additional File 1, Table S4.

### Estimates of Patient Prevalence

To estimate the expected frequency of high chromosome number in the patient population, we reviewed the Mitelman Database of Chromosome Aberrations in Cancer [20].

### Transcriptomics

mRNA transcript expression was quantified by using the Affymetrix U133 Plus2 GeneChips in triplicate. First, cell lines were plated in triplicate and lysed in TRIzol. Lysates were captured with chloroform and purified using QIAGEN RNeasy Mini Kit (QIAgen, Inc., Valencia, CA). cDNA was prepared from 5 μg total RNA using the Invitrogen SuperScript Double-Stranded cDNA Synthesis Kit (Invitrogen, Inc, Carlsbad, CA) and amplified using the ENZO BioArray High-Yield RNA Transcript Labeling Kit (Enzo Biochem, Inc. New York, NY). Finally, the samples were fragmented and hybridized to the HG-U133Plus2 GeneChips, stained and scanned according to the manufacturer's protocols. Transcript abundance was estimated by normalizing all probe signal intensities were normalized to a value of 150 using the mas5 algorithm in the Affymetrix Microarray Analysis Suite 5.0. For subsequent analysis, the average probe intensity was used for triplicates. Values of mRNA abundance for Aurora A, B and C are presented in Additional File 1, Table S4.

### Kinase Screening

Enzymatic kinase screening assays for GSK7160916 were performed by the Upstate Group http://www.upstate.com using the KinaseProfiler to determine activity across a range of kinases including the ABL kinase oncogene.

## Results

### In Vitro Response Data

**Figure 1:**
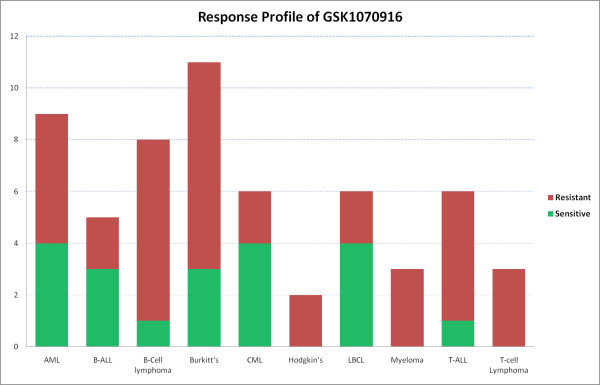
**Response profile of GSK1070916 for hematological cell lines using cell cycle analysis and cell death measures to determine sensitivity and resistance**. Cell lines that are early and moderate responders by cell cycle analysis with a Ymin/T0 ratio ≤ 0.5 were considered sensitive (see METHODS).

Based on proliferation, most of the hematological cell lines were responsive to GSK1070916 with a median EC50 of 7 nM. Since cancer cell death is a more desired phenotype, the *in vitro *response of 91 hematological cell lines were defined based on both time of response and degree of cell death. 20/91 (22%) cell lines were designated sensitive and 39/91 (43%) cell lines were designated resistant (where sensitive and resistant is defined in the Methods). Discordant values between proliferation and cell death were identified for 32 cell lines and subsequently excluded, leaving 59 cell lines in the panel for further analysis. The response of CML (4/6, 67%), Large B-Cell lymphomas (4/6, 67%) and B-Cell Acute lymphocytic leukemia (4/6, 67%) subtypes were among the more sensitive subtypes. Conversely, T-cell Acute lymphoblastic leukemia (1/6, 17%) B-cell lymphomas (1/8, 13%) and Myelomas (0/3, 0%) were more resistant among the different subtypes. (Figure 1; Additional File 1, Table S1).

### Modal Chromosome Number

**Figure 2:**
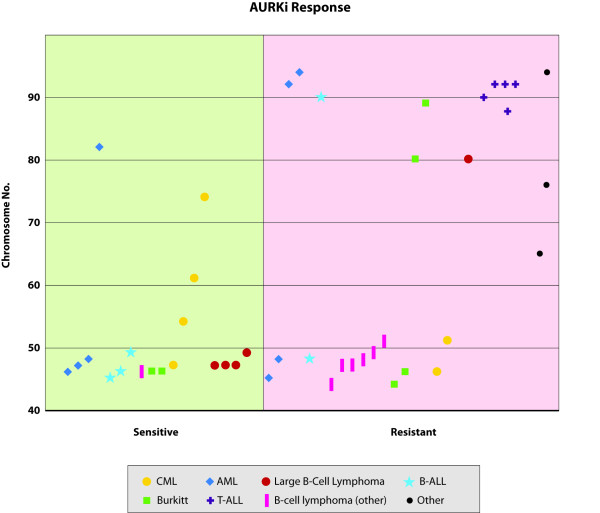
**Response vs. Chromosome Number**. Response profile of GSK1070916 for various hematological cell line tumor types (n = 45). Those cell lines that were responsive to treatment are on the left and those that were resistant are on the right. Higher chromosome numbers is more prevalent for the less sensitive phenotypes.

In the analysis of the impact of chromosome number on response, we found that most cell lines that were approximately triploid or greater in chromosome number (3n, > 69) were less sensitive to GSK1070916 (Figure 2). This relationship with high chromosome number and resistant phenotype was apparent in most hematological subtypes, with exception of two cell lines, an AML line (HL-60) and a CML line (EM-2). Notably, three CML lines with hyperdiploidy (>2n) and hypertriploidy (>3n) still showed sensitive response (HL-60, EM-2, KU-812). In addition to inhibiting Aurora B and C, GSK1070916 also has activity for ABL (Additional File 1, Table S6) which potentially contributes to the sensitivity observed in these cell lines.

Comparison of the two response phenotypes for modal chromosome number, using a chromosome count of (3n) as the cutoff, showed a difference in the response between the two cell line populations (p-value = 0.0098, two-tailed Fisher Exact Test; Table 1). Using the *in-vitro *data as a model for evaluating diploid chromosome number as potential marker for patient selection provided reasonably high sensitivity in predicting response rates (16/18 = 89%) but a lower specificity in predicting those patients that would not respond to treatment (13/27 = 48%). Not surprisingly, the negative predictive value for low chromosome number was higher (NPV = 14/16 = 88%) compared to the positive predictive value (PPV = 16/33 = 49%).

**Table 1:** Response to GSK107916 among populations of cells with high and low modal chromosome number in a 2 × 2 contingency table.

	Sensitive	Resistant	*Total*
**Diploid (~2n)**	16	13	***33***
**High Modality (>3n)**	2	14	***12***
***Total***	***18***	***27***	***45***

### Polyploidy in Tumor Subpopulations

**Figure 3:**
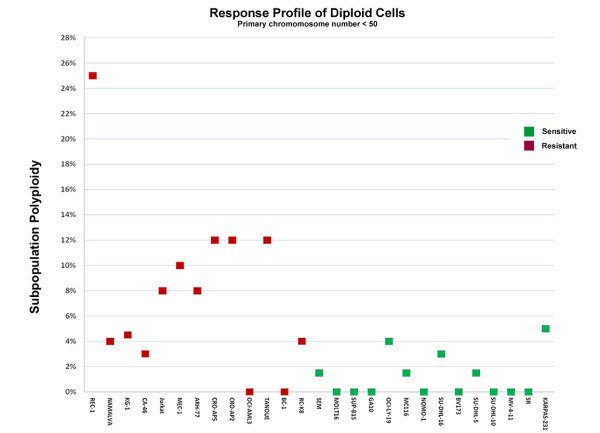
**The response profile of GSK1070916 for cell lines with a primary diploid chromosome number (<50)**. The percentage of polyploidy within subpopulations of these cells is provided on the y axis. Resistant cell lines appeared to have elevated polyploidy among cell subpopulations.

In addition to the data for the primary chromosome number, as used in Figure 2, karyotype data can be reviewed for percentage of polyploidy in cell subpopulations. For instance, the karyotype data for the TANOUE cell line has a chromosome modal number of 48 for the primary population of cells, but also 12% of the cell population was polyploid (See Additional File 1, Table S2 for karyotype data). To evaluate the effect these subpopulations may have on response, we reviewed the ploidy of cell subpopulations for cell lines with *low/diploid *chromosome number (<50) in the primary population (Figure 3). Interestingly, with the limited subset of karyotype data available, we found that the average percentage of polyploid subpopulations was substantially higher for the resistant cell lines compared to sensitive cell lines in the panel. (7.9% vs. 1.2%, n = 28, p-value = 0.00014, Unpaired t-test, 95%, CI 0.0284- 0.1044) (Additional File 1, Table S3).

### GSK1070916 Treatment Generates Polyploid Phenotype

**Figure 4:**
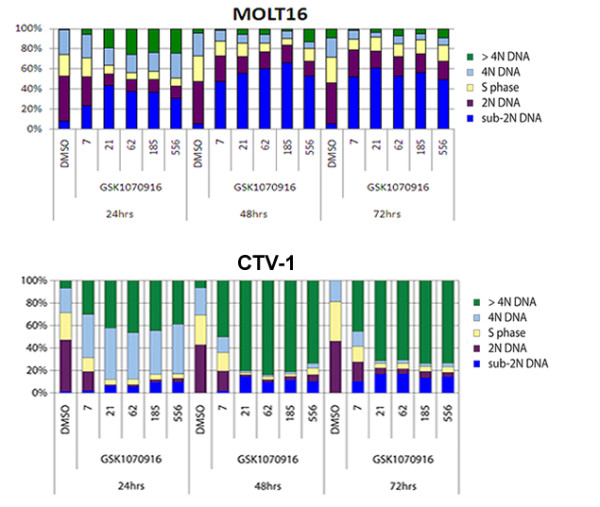
**Cell cycle distribution from fluorescent-activated cell sorting (FACs) analysis of T-ALL cell lines after treatment with GSK1070916 at 24, 46, and 72 hours**. (a) MOLT-16 was sensitive to GSK1070916 and showed increasing amounts of sub-2N DNA (blue) indicating cell death.(b) In contrast, CTV-1 had higher amounts of 4N DNA or greater (light blue, green) which increased with prolonged exposure to GSK1070916, generating a large multinucleated resistant phenotype.

Treatment of cancer cells with GSK1070916 yielded phenotypes with polyploid DNA content resulting from chromosome replication without nuclear or cell division. A sensitive and diploid T-ALL cell line MOLT16, and a polyploid and resistant T-ALL cell line CTV-1 were treated with increasing concentrations of GSK1070916 for different time periods, and a flow cytometry study was performed. For the sensitive cell line MOLT16, a population of polyploid cells emerged within 24 hrs and maintained their growth with increasing drug concentration. However, over longer period of drug treatment (48 hr and 72 hr), the percentage of polyploid cells were significantly reduced, and there was a simultaneous increase of sub-G1 population representing dead cells, suggesting that the polyploid cells developed earlier were not being tolerated and subsequently died. This is in contrast to CTV-1, which exhibited much higher levels of polyploidy cells and low cell death throughout the study. (Figure 4)

### Genetics Analysis

The background genetics of the hematological cell line panel was reviewed in relation to Aurora inhibition by GSK1070916. Expression profiles of Aurora A, B, and C were evaluated in terms of response to Aurora inhibition and no association was observed (p-value = 0.79 and 0.96 respectively, unpaired t-test, Additional File 1, Table S4).

In our response dataset, we observed 6 of the 7 T-ALL cell lines with high chromosome number also had mutations in NOTCH1. To investigate this further, we collected additional mutation data from public databases for T-ALL cell lines (Additional File 1, Table S4). For this dataset, a notable association with NOTCH1 and high modal chromosome number was identified (Table 2, n = 23, p-value 0.0066, two-tailed Fisher Exact Test).

**Table 2:** Association of NOTCH1 mutation status to high modal chromosome number in T-ALL cell lines.

	WT	Mutant NOTCH1	*Total*
**Diploid (~2n)**	7	2	***9***
**High Modality (>3n)**	2	12	***14***
***Total***	***10***	***14***	***23***

### Prevalence of High Chromosome Modality in Patient Population

To estimate the expected frequency of high chromosome modality in a prospective patient population, we reviewed the Mitelman Database of Chromosome Aberrations in Cancer (see METHODS). The most prevalent cases of high chromosome modality were found in Hodgkin's Lymphoma, Myeloma, and B-cell Acute Lymphocytic Leukemia. Conversely, AML and T-cell Acute Lymphoblastic Leukemia subtypes had a lower prevalence of high chromosome modality (Table 3a).

**Table 3:** Estimated frequency of high modality in major hematological patient populations.

Tumor Type	>2n	>3n	Total Cases
AML	4.6%	1.5%	14,611
B-ALL	25.0%	2.0%	3,769
NHL - B-Cell	14.8%	8.2%	3,542
NHL - T-Cell	7.2%	5.1%	1,497
Hodgkins	48.8%	30.3%	244
T-ALL	5.9%	3.5%	1,130
Myeloma	39.8%	8.3%	1,561

For the GSK1070916 inhibitor, one prospective target patient population is Non-Hodgkin's B-cell Lymphoma. To ascertain the relative frequency of high chromosome modality in this patient population, frequency data for each subtype of B-cell lymphoma was collected and reviewed. The distribution of high chromosome modality was varied with Diffuse Large B-Cell, Follicular, and Mantle lymphoma subtypes having higher frequencies compared to Burkitt and MALT NHL subtypes (Table 3b).

## Discussion

Karyotyping is a standard clinical practice for hematological malignancies, and the cytogenetics of the disease not only helps with diagnosis, but often provides prognostic values [21-23]. With karyotype data from these cell lines, we discovered that high chromosome number in cell lines were associated with resistance to GSK1070916. As with other Aurora B inhibitors, treatment with GSK1070916 generally elicited a polyploidy phenotype in cell lines. This suggests cancer cells with a polyploid phenotype might have developed mechanisms to bypass checkpoints for polyploidy and thus are resistant to Aurora inhibition. Our comprehensive review of publicly available karyotype data revealed subtypes of hematological malignancies with high frequencies of polyploidy. Conveniently, it is standard clinical practice to perform karyotyping on hematological cancer cells and chromosome number can serve as an attractive resistance marker for patient response enrichment for GSK1070916 in malignancies such as NHL.

A number of Aurora kinase inhibitors are already in clinical or preclinical development including GSK1070916, VX-680, AZD1152, PHA-739358, AT9283 and CYC116 [24-28]. Aurora kinase Inhibitors have shown potential efficacy for a variety of hematological tumor subtypes including AML, ALL and CML [29-33]. As with other targeted therapies, predictive biomarkers for GSK1070916 that could stratify patient populations can accelerate clinical development and cell line models have proven to be useful system for this purpose [34]. However, most of the hematological cell lines in our panel exhibited high sensitivity using proliferation as a measure of response. This sensitive response profile is likely due to the continuous proliferating nature of the established cell lines in tissue culture. Since cancer cell death is a more desired response in clinic, measures of cell death were used as the criteria to categorize response to GSK107016.

Using these criteria, our cell line panel exhibited sensitivity with GSK1070916 in a broad range of leukemias (AML, B-ALL, and CML) and two subtypes of NHL (Burkitt's, Large B-Cell Lymphoma). These findings are generally consistent with response profiles observed with other Aurora inhibitors [29,31,33] and suggests these disease subtypes can serve as important predictors of response.

Genetic and cytogenetic information for the cell lines were used to discover genetic markers with predictive value. Cell lines with the polyploid phenotype were associated with resistance to GSK1070916. This observation was particularly striking in the response profile for T-ALL cells in which a majority of cells (5/6) had both high chromosome number and resistance to GSK1070916 with the sensitive cell line (MOLT-16) also having the low chromosome phenotype. Not surprisingly, three CML lines with hyperdiploidy (>2n) and hypertriploidy (>3n) still maintained a sensitive response profile. The sensitivity observed in CML cell lines, even with the polyploid phenotype, was not unexpected since GSK1070916 inhibits ABL, and aurora kinase inhibitors that also inhibit ABL can be considered a potential therapeutic alternative for patients resistant to Imatinib [35].

Cell lines and tumors can often exhibit heterogeneous genetic backgrounds from diverse subpopulations. Upon examination of the cell lines with low primary chromosome number, we found a higher proportion of polyploidy among cell subpopulations in the resistant group. For instance, in our panel of B-cell lymphoma cell lines, 6 of the 7 cell lines were resistant to GSK1070916 and contained low chromosome number in the primary population of cells. However, when in reviewing the ploidy content in the cell subpopulations in this tumor type, we observe high ploidy content in numerous B-cell lymphoma lines (e.g. REC-1, 25% polyploidy). This further underscores the significance of the general observation between polyploidy and resistance. For these data, we hypothesize there is a selective growth advantage for the subpopulation of cells with the polyploid phenotype during Aurora inhibition. This may represent a resistance mechanism that potentially can develop upon prolonged drug treatment with Aurora inhibitors. These findings warrant further investigation about the relationship of chromosome number in primary and secondary populations of the tumor during and after treatment to monitor potential evolving resistance.

Inhibition of Aurora B does not inhibit cell cycle progression but rather enters and exits mitosis with normal kinetics, with cells re-replicating their genome [36]. Treatment of cancer cells with GSK1070916 typically yields a polyploid phenotype resulting from chromosome replication without nuclear or cell division. Our FACS analysis of GSK1070916 treatment shows that for sensitive cells, polyploid cell populations would develop during earlier time points and would be killed upon longer drug incubation. For resistant cell lines, however, polyploid cell populations were tolerated over time and significantly less cell death was observed. To maintain genome integrity, cells generally have developed mechanisms/checkpoints to prevent polyploidy [37]. It can be hypothesized that for cells that are primarily polyploid, they have developed mechanisms to bypass these checkpoints to tolerate polyploidy and therefore can evade cell death by AURKB/C inhibition. One of these mechanisms could be p53 dependent tetraploidy checkpoint [38-40]. Interestingly, excluding cell lines with high chromosome content (chromosome number >50 or polyploidy in >5% of cell population), 4/5 sensitive lines were reported wild-type for p53 while 3/4 resistant lines were p53 mutant (Additional File 1, Table S5). These data further suggests that inactivation of polyploidy checkpoints might contribute to resistance during AURKB inhibition.

The expression profile for Aurora B and C in our panel did not show any relationship with response to GSK1070916 (Additional File 1, Table S4). However, since the expression data in our panel does not reflect the relative expression of the Aurora genes at the time of mitosis, the relationship of Aurora expression and response to GSK1070916 is still unclear. In a subsequent analysis of the background genetics, we found NOTCH1 mutation status to be associated with high chromosome number in T-ALL cells. In concordance with these findings, 3 of 4 resistant T-ALL cell lines with polyploidy also had mutations in NOTCH1. While there was one AML cell line (ML-2) with a NOTCH1 mutation which appeared to be tetraploidy and was resistant to GSK1070916, a majority of cell lines that were not T-ALL cell lines were wild-type for NOTCH1. Since the association of NOTCH1 mutation status with response to GSK1070916 was beyond the scope of this study, no further data was collected to fully confirm this relationship. While NOTCH activation has been reported to be associated with tetraploidy and chromosomal instability in meningiomas [41], the specific mechanism by which these mutations may play in the formation of the observed polyploid phenotype in T-ALL cells has yet to be determined. Interestingly, NOTCH signaling has also been considered to play a role in cancer stem cell regulation [42] but it is unclear what role the polyploid phenotype may play for these cell types.

Estimates of patient prevalence for a biomarker are critical for determining the appropriate patient selection strategy. These estimates of prevalence can provide guidance on the number of patients needed to screen for the marker and the subtypes of the disease that are most likely to provide a positive or negative response. The prevalence of the high modal chromosome number in patients can be estimated using cytogenetic data publicly available from the Mitelman database. We found the frequency of high chromosome number is generally higher among lymphoma compared to leukemia malignancies. While the Hodgkin's lymphoma subtype has an elevated frequency of high chromosome modality in its patient population, the NHL subtypes represent a population of patients with a significant unmet medical need. Further review of NHL subtypes showed that Follicular and Diffuse Large B-Cell are the most promising as candidate NHL subtypes for using high chromosome number as a marker of negative response to Aurora inhibition. A review of NOTCH mutations in the COSMIC database [19] for T-ALL tumors show a mutation frequency of 40% suggesting that T-ALL may also be a potentially attractive subtype for patient stratification.

## Conclusions

Identification of cytogenetic abnormalities using karyotyping for prognosis and treatment of hematological malignancies has been a standard diagnostic tool for many years [43-46]. Detection of polyploidy in cells, with its ease of measurement, low costs, and biological relevance as a negative predictor of response to Aurora inhibition, can be a powerful tool to enrich patients that can potentially respond to GSK1070916.

## Competing interests

The authors declare that they have no competing interests.

## Authors' contributions

CAO, RP carried out the cell cycle and response studies. CM participated in the design of the study and performed the statistical analysis. YD, MAH, CM conceived of the study, and participated in its design and coordination and helped to draft the manuscript. All authors read and approved the final manuscript.

**Table 4:** Prevalence of high modality in NHL B-Cell Lymphoma subtypes.

NHL Subtype	>2n	>3n	Total Cases
Diffuse Large	27.5%	13.7%	1225
Follicular	18.3%	8.0%	1330
Mantle	9.7%	7.7%	402
Burkitt	6.4%	2.0%	659
MALT	5.9%	3.5%	340

## Supplementary Material

Additional file 1 **Additional Table S1**. Response Data for treatment of cells with GSK1070916. Response is designated through evaluation of Cell Cycle Analysis (FACs), Ymin/T0 and EC50 values (See METHODS). **Additional Table S2**. Available Karyotype data for Cell lines treated with GSK1070916. **Additional Table S3**. Among cell lines with low native modal chromosome number (< 50), the estimated polyploidy in the subpopulation of cells are reviewed in terms of response to Aurora inhibition by GSK1070916. **Additional Table S4**. Background Genetics data for Cell lines treated with GSK1070916. **Additional Table S5**. Review of Cell lines in panel with low native chromosome number (< 50) and low polyploid in subpopulations (< = 5%). **Additional Table S6**. Percent inhibition from Kinase screen of GSK1070916 for human and mouse ABL oncogene at 0.3 uM and 10 uM Click here for file
